# Supplementary material for: Single versus dual antiplatelet therapy following peripheral arterial endovascular intervention for chronic limb threatening ischaemia: Retrospective cohort study
Source: PLoS One. 2020 Jun 11;15(6):e0234271. doi: 10.1371/journal.pone.0234271 (PMC7289358; doi:10.1371/journal.pone.0234271)
Supplement: S1 Table — (DOCX) [file pone.0234271.s001.docx]

**Supplementary Table 1A**: Multivariate Analysis with confounder correction for amputation free survival

| **Risk Factor** | **HR (95% CI)** | ***p*** |
| --- | --- | --- |
| White cell count | 1.1(1.01-1.1) | 0.003 |
| Haemoglobin | 1.0(0.98-1.0) | 0.03 |
| Urea | 1.1(1.05-1.11) | 4.83e-07 |
| Albumin | 0.94(0.92-0.96) | 4.20e-07 |
| Anti-platelet | 0.94(0.74-1.35) | 0.7 |
| CCF* | 1.3(0.97-1.68) | 0.09 |
| CVE** | 1.5(1.1-2.02) | 0.01 |
| COPD^a^ | 1.4(1.08-2.02) | 0.02 |
| Hypertension | 1.4(1.04-1.92) | 0.03 |
|  |  |  |
| **Anatomy** | **HR (95% CI)** | ***p*** |
| Femoro-popliteal | 1.4(1.08-1.91) | 0.01 |
| Below the knee | 1.8(1.35-2.53) | 0.00013 |
|  |  |  |
|  | **HR (95% CI)** | ***p*** |
| Stent placement | 1.5(1.03-2.19) | 0.03 |

*CCF = Congestive Cardiac Failure

**CVE = Cerebrovascular Event (including stroke and transient ischaemic attack)

^a^COPD = Chronic Obstructive Pulmonary Disease

Confounder correction using Cox Proportional Hazards method was undertaken with stepwise selection of confounders through minimization of the Akaike Information Criterion to reduce model over-fitting.

Factors considered but rejected by the process: ischaemic heart disease, diabetes mellitus, creatinine, eGFR, platelet count, statin use and angiotensin converting enzyme inhibitor or angiotensin II receptor blocker use.

Anatomical location considered but rejected: aorto-iliac segment

**Supplementary Table 1B**: Multivariate Analysis with confounder correction for amputation free survival truncated at 12 months

| **Risk Factor** | **OR (95% CI)** | ***p*** |
| --- | --- | --- |
| Creatinine | 1.0(0.99-1.0) | 0.11 |
| Albumin | 0.95(0.92-0.98) | 0.00425 |
| Anti-platelet | 0.8(0.5-1.2) | 0.3 |
| Statin | 1.6(1.0-2.6) | 0.05 |
| IHD* | 0.66(0.43-1.0) | 0.05 |
| Diabetes mellitus | 1.5(0.95-2.2) | 0.08 |
| COPD^a^ | 1.6(0.94-2.7) | 0.08 |
|  |  |  |
| **Anatomy** | **OR (95% CI)** | ***p*** |
| Below the knee | 1.6(1.0-2.4) | 0.05 |

*IHD = Ischaemic Heart Disease

^a^COPD = Chronic Obstructive Pulmonary Disease

Confounder correction using logistic regression was undertaken with stepwise selection of confounders through minimization of the Akaike Information Criterion to reduce model over-fitting.

Factors considered but rejected by the process: white cell count, haemoglobin, platelet count, urea, eGFR, platelet count, angiotensin converting enzyme inhibitor or angiotensin II receptor blocker use, congestive cardiac failure, cerebrovascular accident and hypertension.

Anatomical location considered but rejected: aorto-iliac segment, femoro-popliteal segement

Endovascular device: stent placement was also rejected by the process

**Supplementary Table 1C**: Multivariate Analysis with confounder correction for survival

| **Risk Factor** | **HR (95% CI)** | ***p*** |
| --- | --- | --- |
| Haemoglobin | 1.0(0.98-1.0) | 0.0066 |
| Urea | 1.1(1.04-1.11) | 9.85e-06 |
| Albumin | 0.94(0.92-0.97) | 1.99e-05 |
| Anti-platelet | 1.0(0.74-1.4) | 0.9 |
| IHD* | 1.3(1.0-1.8) | 0.05 |
| CCF** | 1.6(1.2-1.68) | 0.09 |
| CVE^a^ | 1.5(1.1-2.2) | 0.0046 |
| COPD^b^ | 1.4(1.03-2.1) | 0.0299 |
|  |  |  |
| **Anatomy** | **HR (95% CI)** | ***p*** |
| Femoro-popliteal | 1.4(1.05-2.01) | 0.02 |
| Below the knee | 1.7(1.17-2.4) | 0.0049 |
|  |  |  |
|  | **HR (95% CI)** | ***p*** |
| Stent placement | 1.9(1.2-2.9) | 0.003 |

* IHD = Ischaemic Heart Disease

**CCF = Congestive Cardiac Failure

^a^CVE = Cerebrovascular Event (including stroke and transient ischaemic attack)

^b^COPD = Chronic Obstructive Pulmonary Disease

Confounder correction using Cox Proportional Hazards method was undertaken with stepwise selection of confounders through minimization of the Akaike Information Criterion to reduce model over-fitting.

Factors considered but rejected by the process: white cell count, platelet count, creatinine, urea, eGFR, diabetes mellitus, hypertension, statin use and angiotensin converting enzyme inhibitor or angiotensin II receptor blocker use.

Anatomical location considered but rejected: aorto-iliac segment

**Supplementary Table 1D**: Multivariate Analysis with confounder correction for limb salvage

| **Risk Factor** | **HR (95% CI)** | ***p*** |
| --- | --- | --- |
| White cell count | 1.1(1.01-1.2) | 0.003 |
| Creatinine | 1.0(0.99-1.0) | 0.093 |
| eGFR | 1.0(1.0-1.02) | 0.06 |
| Albumin | 0.95(0.92-0.99) | 0.010 |
| Anti-platelet | 0.86(0.4-1.6) | 0.6 |
| Statin use | 1.4(0.85-2.5) | 0.17 |
| IHD* | 0.64(0.4-1.02) | 0.06 |
| Diabetes mellitus | 1.5(0.89-2.6) | 0.12 |
| COPD^a^ | 1.6(0.89-2.73) | 0.119 |
| Hypertension | 2.5(1.29-5.16) | 0.007 |
|  |  |  |
| **Anatomy** | **HR (95% CI)** | ***p*** |
| Below the knee | 1.5(0.91-2.54) | 0.107 |
|  |  |  |
|  | **HR (95% CI)** | ***p*** |
| Stent placement | 0.56(0.29-1.1) | 0.082 |

*IHD = Congestive Cardiac Failure

^a^COPD = Chronic Obstructive Pulmonary Disease

Confounder correction using Cox Proportional Hazards method was undertaken with stepwise selection of confounders through minimization of the Akaike Information Criterion to reduce model over-fitting.

Factors considered but rejected by the process: haemoglobin, platelet count, urea, creatinine, eGFR, platelet count, cerebrovascular event, congestive cardiac failure and angiotensin converting enzyme inhibitor or angiotensin II receptor blocker use.

Anatomical location considered but rejected: aorto-iliac segment and femoro-popliteal segment
