# Supplementary material for: Single versus dual antiplatelet therapy following peripheral arterial endovascular intervention for chronic limb threatening ischaemia: Retrospective cohort study
Source: PLoS One. 2020 Jun 11;15(6):e0234271. doi: 10.1371/journal.pone.0234271 (PMC7289358; doi:10.1371/journal.pone.0234271)
Supplement: Attachment — Submitted filename: PLOS DAP review.docx [file pone.0234271.s002.docx]

Re: Single versus dual antiplatelet therapy following peripheral arterial endovascular intervention for chronic limb threatening ischaemia: retrospective cohort study

Manuscript Draft-- Manuscript Number: PONE-D-20-04980

Reviewers comments

An interesting and timely manuscript. I have a number of comments.

1. Any study looking at PAD patients is difficult and results tempered by the heterogeneity seen within the population – especially as the authors have included aorto-iliac / femoropopliteal and infrapopliteal endovascular revasculairsation in this dataset. Decision making around the role of antiplatelet therapy can be nuanced and often involves lesion type / endovascular result / run off / patient risk of bleeding – especially given a lack of antiplatelet therapy protocol within the department. To this end such data would be useful to allow further interpretation of the results. Specifically, eg TASC lesion criteria treated, run off – CFA / SFA / PFA disease of AI treatment and crural run off for FP treated lesions. Of more importance is risk of bleeding for an older and frail patient group as this may well have influenced decision making eg use of PPI / H2 antagonists at the time of revascularisation as a proxy for GI bleed risk or a HASBLEED 2 score. I am also surprised that allergy to aspirin / other antiplatelet therapies wasn’t an exclusion criterion.
2. The authors admit that there is in built bias with regard to decision making around dual antiplatelet therapy – was there a difference between surgeons and radiologists. While managing the change in antiplatelet strategy is straightforward if the patient is an in patient what was the logistical arrangement for those patient attending as an out patient. Was the patient sent home with a prescription or was this left in the hands of the GP with a discharge letter – this is important as planned antiplatelet management does not equate to initiation of therapy / adherence. Adherence is a whole different ball game which this study would not be able to determine and has to be accepted as a weakness of the study.
3. The trend of change in AP prescription does cloud the data and it may be that the use of DAP is better but this hasn’t been seen yet as the effect are seen out to 2 o r3 or 4 years. Could the authors comment on this.
4. There are a number of assumptions around the sample size calculation which need to be mentioned in the discussion.
5. It is not well signposted in the manuscript that claudicants were excluded. Further is the data looking at per limb or per patient. Did the fact that a patient had two limbs treated alter the management of antiplatelet regime. Eg. If the ipisilateral limb was treated and patient put on DAP, and the the contra limb treated – was this patient then excluded as they were already on dual antiplatelet therapy? If the same patient was initially on a single AP agent and then after the second procedure put on DAP in which group were they classed? Would the data be cleaner if only those patients having one limb treated were included.
6. The authors need to define the comorbidities – ie. What is actually meant by ischaemic heart disease – by having detailed patients notes I am sure that they have strict definitions for inclusion.
7. Which variables were excluded with data missing of >15%. Which of the variable used had significant data missing eg>5%?
